# Coffee and caffeine intake in relation to symptoms of psychological disorders among adults

**DOI:** 10.1017/S1368980022000271

**Published:** 2022-12

**Authors:** Saeedeh Nouri-Majd, Asma Salari-Moghaddam, Ammar Hassanzadeh Keshteli, Hamid Afshar, Ahmad Esmaillzadeh, Peyman Adibi

**Affiliations:** 1 Department of Community Nutrition, School of Nutritional Sciences and Dietetics, Tehran University of Medical Sciences, Tehran 14155-6117, Iran; 2 Department of Medicine, University of Alberta, Edmonton, AB, Canada; 3 Integrative Functional Gastroenterology Research Center, Isfahan University of Medical Sciences, Isfahan, Iran; 4 Department of Psychiatry, Psychosomatic Research Center, Isfahan University of Medical Sciences, Isfahan, Iran; 5 Obesity and Eating Habits Research Center, Endocrinology and Metabolism Molecular – Cellular Sciences Institute, Tehran University of Medical Sciences, Tehran, Iran; 6 Department of Community Nutrition, School of Nutrition and Food Science, Isfahan University of Medical Sciences, Isfahan, Iran

**Keywords:** Coffee, Caffeine, Depression, Anxiety, Psychological distress, Psychological disorders

## Abstract

**Objective:**

Given that there is an inconsistency in the findings related to the relationship between coffee and caffeine consumption and symptoms of psychological disorders, we performed a cross-sectional analysis to examine the association between coffee and caffeine intake and symptoms of psychological disorders among adults.

**Design:**

In this cross-sectional study, 3362 participants were included. We assessed the coffee and caffeine intakes using a self-completed FFQ. Symptoms of depression, anxiety and psychological distress were assessed using Hospital Anxiety and Depression Scale and General Health Questionnaire screening tools.

**Setting:**

Fifty different healthcare centres located in the province of Isfahan, Iran.

**Participants:**

This study was performed on 3362 Iranian general adults working in healthcare centres.

**Results:**

The mean age of participants in this study was 36·2 ± 7·8 years. After controlling for potential confounders, individuals who consumed coffee weekly or more had a significantly lower odds of symptoms of depression (OR 0·67; 95 % CI (0·46, 0·96)) and symptoms of anxiety (OR 0·57; 95 % CI (0·34, 0·95)) compared with those who did not consume coffee. However, no significant association was found between coffee intake and symptoms of psychological distress (OR 0·98; 95 % CI (0·68, 1·42)). No significant relationship was found between caffeine intake and odds of symptoms of depression (OR 0·94; 95 % CI (0·75, 1·16)), symptoms of anxiety (OR 0·90; 95 % CI (0·67, 1·20)) and symptoms of psychological distress (OR 1·13; 95 % CI (0·89, 1·42)).

**Conclusion:**

Compared with lack of coffee intake, weekly or more coffee consumption might be correlated with symptoms of depression and anxiety.

The increasing trend of mental health disorders in the world has gained great attention^(1)^. Depression and anxiety are the most common psychological disorders in the world that are associated with disability and comorbidity^(2)^. In Iran, the prevalence of mental disorders has been reported at approximately 23 %^(3)^.

Diet has long been associated with mental health. Coffee is a common drink in most parts of the world^(4)^. Evidence for the association between coffee and caffeine intake odds of psychological disorders is limited^(5)^. Some previous studies have examined this association; however, their findings remain inconsistent^(6,7)^. Most studies have found an inverse association between coffee and caffeine intake and risk of mental disorders^(8–11)^. This was also confirmed by a cohort study, within which a linear inverse association between coffee consumption and risk of depression symptoms was reported^(12)^. However, some studies reached no clear association of coffee or caffeine intake with depression and anxiety^(13,14)^. Some others have even reported an increased risk of depression by coffee or caffeine consumption^(15,16)^.

Most studies on the association between coffee and caffeine intake and mental health come from Western countries and little information is available from developing countries. Given the nutrition transition in developing countries, changing the pattern of drinking from tea consumption to coffee consumption, along with the high prevalence of depression and anxiety in these countries, assessment of the contribution of coffee and caffeine intake to these conditions is of high importance. It must also be kept in mind that most studies on these relationships did not adjust for many potential confounders that might affect mental health, especially for dietary intakes that can confound the association between coffee and caffeine intake and symptoms of psychological disorders. To our knowledge, no study have been conducted on the association between coffee and caffeine consumption and odds of symptoms of psychological disorders in the general population in the Middle East. Therefore, this study aimed to investigate the association between coffee and caffeine intake and odds of symptoms of psychological disorders including anxiety, depression and psychological distress in the Iranian adult population.

## Methods and materials

### Participants

This study was based on a comprehensive cross-sectional study, named Study on the Epidemiology of Psychological, Alimentary Health and Nutrition (SEPAHAN), in fifty different medical centres throughout Isfahan province, which was affiliated to Isfahan University of Medical Sciences (IUMS). Detailed information about the SEPAHAN project has been published elsewhere^(17)^. Data for this study were collected in two main phases between April 2010 and May 2010. A total of 4763 people completed the questionnaires in both phases. In this study, people who had a total daily energy intake of less than 800 or more than 4200 kJ/d were excluded^(18)^, as well as those who completed the questionnaire incompletely and did not have some demographic, anthropometric, dietary or psychological information. Therefore, 3362 participants were included in the analysis of this study, who had complete information about dietary intakes and psychological characteristics. The participant’s flowchart for this study has been published in previous articles^(19)^. Written consent forms were given to participants and all of them submitted the form.

### Assessment of dietary intake

In this study, all dietary information was gathered through the use of a Willett-format 106-item semi-quantitative FFQ (DS-FFQ). The investigators of the SEPAHAN study had developed this questionnaire specifically for Iranian adults^(20)^. In this questionnaire, there were five categories of dishes and foods: (1) fruits and vegetables (twenty-two items); (2) mixed dishes (canned or cooked, twenty-nine items); (3) dairy products (butter, dairies and cream, nine items); (4) grains (cakes, potato, biscuits and different types of bread, ten items) and (5) beverages and miscellaneous food items (including beverages, sweets, desserts, fast foods and nuts, thirty-six items). To calculate the amounts of foods consumed, the booklet of ‘household measures’ was used^(21)^.

Validation of the questionnaire was examined in a study on 200 randomly selected people. These subjects were requested to fill the questionnaire twice, at the beginning of the study and 6 months later. Three detailed dietary records were also completed by subjects. Comparison of data from the questionnaire and the average of food diaries revealed that the questionnaire works well to estimate long-term dietary intakes^(20)^.

### Calculation of coffee and caffeine intake

Coffee consumption was assessed based on the average number of coffee cups that participants typically consumed in the previous year. Participants could choose from one of the following frequency response categories: ‘never or more than 1 cup/month’, ‘1–3 cups/month’, ‘1 cup/week’, ‘2–4 cups/week’, ‘5–6 cups/week’, ‘1 cups/d’, ‘2–3 cups/d’, ‘4–5 cups/d’ and ‘6 cups or more in a day’. Coffee is not consumed regularly by most people in Iran and is not a common beverage, so we put people in the categories of none, monthly (1–3 cups/month), weekly or more (1–6 cups/week, 1-more than 6 cups/d) based on coffee consumption. Total caffeine intake was estimated by summing up the caffeine that participants took from all caffeine-containing foods and beverages (types of chocolate, cocoa, tea, soft drink and coffee). For each cup of coffee intake in the current study, we considered the caffeine levels as 96 mg, as mentioned in the USDA food table^(22)^. Participants were categorised into tertiles based on their caffeine intake (<57·4 mg/d, 57·4–103·4 mg/d and ≥103·5 mg/d).

### Assessment of psychological proﬁle

In this study, the Iranian version of the Hospital Anxiety and Depression Scale (HADS) was used to screen for symptoms of anxiety and depression^(23)^. HADS could be a concise and useful questionnaire to evaluate symptoms of psychological disorders and the symptom severity of depression and anxiety disorders. Although the name of this questionnaire seems to be a hospital-based questionnaire, this questionnaire is designed to screen symptoms of depression and anxiety in normal populations and earlier studies have also used this questionnaire for similar purposes. There are published validation studies of this questionnaire that examined its validity and reliability among both healthy and diseased populations^(23,24)^. The HADS contains two subscales: anxiety and depression and includes fourteen items. Each item consists of a four-point scale; with increasing scores, the level of anxiety and depressive symptoms also increases and 21 is the maximum score for anxiety and depression. In the current study, people with scores of 0–7 were considered ‘normal’ and people with scores of 8 or more on both scales were considered as psychological disorders. The convergent validation of translated version of the HADS questionnaire was examined in 167 Iranian adults using the correlation of each item with its hypothesised scale. Pearson’s correlation coefficients varied from 0·47 to 0·83 (*P* < 0·001) for anxiety subscale and from 0·48 to 0·86 (*P* < 0·001) for the depression subscale, indicating that the questionnaire provides relatively valid measures of psychological health^(25)^. In this study, we used a valid Iranian version of the General Health Questionnaire (GHQ) with twelve items to assess psychological distress^(26)^. At GHQ-12, participants are asked whether they have recently experienced certain symptoms of psychological distress or have changed their behaviour. So, this questionnaire is a short and easy tool for measuring current and primary mental health. In this questionnaire, for each item, there is a four-point scale including much more than usual, rather more than usual, no more than usual or less than usual. There are two common methods for scoring including bimodal (0-0-1-1) and Likert (0-1-2-3), which give a total score of 12 or 36, respectively. The bimodal scoring method was used in this study. In this study, we defined a score of 4 or more as psychological distress^(27)^. The convergent validity of GHQ-12 was examined in 748 Iranian young people. Signiﬁcant inverse correlation was seen between the GHQ-12 and global quality of life scores (*r* = -0·56, *P* < 0·001)^(26)^.

### Assessment of other variables

Required information on other variables including sex, age, the existence of a chronic condition (asthma, diabetes, colitis, cancers, stroke, heart failure and myocardial infarction), education, marital status, antidepressant use, smoking status and supplements use (minerals, Fe, Ca and vitamins) obtained through demographic and medical history questionnaires. The General Physical Activity Questionnaire (GPPAQ) was used to assess participants’ physical activity levels^(28)^. In this study, participants were classified into two groups: physically inactive (<1 h/week) and physically active (≥1 h/week). The self-report questionnaire was used to collect anthropometric data of participants including height, waist circumference and weight. The validity of self-reported waist circumference, height and weight were examined in a pilot study on 200 participants from the same population. A comparison was made between the self-reported values of anthropometric indices and the measured values in the validation study. The correlation coefficients for self-reported weight, height and waist circumference *v*. corresponding measured values were 0·95 (*P* < 0·001), 0·83 (*P* < 0·001) and 0·60 (*P* < 0·001), respectively. BMI was calculated by dividing weight (kg) by height (m^2^). The correlation coefficient for computed BMI from self-reported values and the one from measured values was 0·70 (*P* < 0·001)^(29)^.

### Statistical analysis

General characteristics of study participants across categories of coffee and caffeine intake were expressed as means and standard deviations for continuous variables and percentages for categorical variables. Dietary intakes of study participants across categories of coffee and caffeine intake were compared using ANOVA. To estimate the OR and 95 % CI for the presence of symptoms of psychological disorders in crude and multivariable-adjusted models, binary logistic regression was used. In these analyses, total energy intake (continuous), age (continuous) and sex (female/male) were controlled in the first model. Further adjustments were made for education (university graduate or diploma/under-diploma), presence of a chronic condition (no/yes), vitamin supplements use (no/yes), smoking status (current smokers, non-smoker and former smokers), antidepressant use (no/yes), physical activity (≥1 h/week/ <1 h/week) and marital status (married/single) were controlled for in the second model. Additional adjustments for *n*-3, dietary fibre intake (continuous), B vitamins and tryptophan (all as continuous) were done in the third model. BMI (as kg/m^2^) was controlled for in the last model. P_for trend_ was determined by considering categories of coffee and caffeine intake as ordinal variables in the logistic regression analysis.

## Results

In the whole population, the prevalence of symptoms of depression, psychological distress and anxiety were 28 % (*n* 943), 22·6 % (*n* 760) and 13·3 % (*n* 448), respectively.

General characteristics of study participants across categories of coffee and caffeine intake are shown in Table 1. Compared with those who did not consume coffee, those who consumed coffee weekly or more were more likely to be vitamin supplements use (*P* < 0·001), physically active (*P* = 0·01), current smokers (*P* = 0·02), university graduated (*P* < 0·001) and less likely to have a chronic condition (*P* = 0·01). No other signiﬁcant differences were seen in terms of other variables. Compared with those in the bottom tertile of caffeine intake, participants in the top tertile of caffeine intake were older (*P* < 0·001), had higher BMI (*P* = 0·05) and were more likely to be university graduates (*P* = 0·01). No significant differences were observed in terms of other variables across tertiles of caffeine intake.

**Table 1:** General characteristics of study participants across categories of coffee and caffeine intake*

	Coffee intake			*P*-value†	Caffeine intake			*P*-value†
	None	Monthly	Weekly or more		T_1_ (<57·4 mg/d)	T_2_ (57·4–103·4 mg/d)	T_3_ (≥103·5 mg/d)	
	%	%	%		%	%	%	
Total sample (*n* 3362)	*n* 2584	*n* 491	*n* 287		*n* 1120	*n* 1121	*n* 1121	
Age (years)								
Mean	36·02	36·51	35·53	0·26	35·66	36·03	37·18	<0·001
s d	7·6	8·3	7·2		8·2	7·6	7·6	
BMI (kg/m^2^)								
Mean	24·87	24·59	24·75	0·32	24·66	24·85	25·19	0·05
s d	3·8	3·7	3·4		3·7	3·8	3·8	
Female	58·8	57·6	64·8	0·11	57·1	61·6	56·1	0·21
Married	82·0	78·7	79·2	0·15	80·8	82·4	81·8	0·60
Antidepressant use	5·7	4·9	4·2	0·46	5·0	6·3	5·4	0·36
Vitamin supplements use	28·4	32·8	39·0	<0·001	29·8	29·9	30·3	0·96
Current smokers	12·2	15·3	16·7	0·02	12·1	13·6	15·8	0·36
Physically active (≥1 h/week)	12·0	16·9	12·5	0·01	12·9	11·9	14·7	1·31
Education (university graduate)	61·2	68·8	75·6	<0·001	58·8	62·4	64·5	0·01
Existence of a chronic condition	4·9	2·0	4·5	0·01	5·2	3·9	4·8	0·34

* All values are mean and standard deviation unless indicated.

† ANOVA for continuous variables and chi-squared test for categorical variables.

Dietary intakes of study participants across categories of coffee and caffeine intake are provided in Table 2. Participants who consumed coffee weekly or more had higher intakes of protein, carbohydrate, fat, energy, dietary fibre, vitamin B_12_, vitamin B_6_, vitamin B_5_, vitamin B_3_, vitamin B_2_, Fe, Zn, tryptophan (*P* < 0·001) and folate (*P* = 0·02) compared with those who did not consume coffee. Participants in the top tertile of caffeine intake had lower intakes of protein, carbohydrate, fat, energy, group B vitamins, Fe, Ca, Zn and tryptophan (*P* < 0·001) than those in the bottom tertile.

**Table 2:** Dietary intakes of study participants across categories of coffee and caffeine intake

	Coffee intake						*P*-value*	Caffeine intake						*P*-value*
	None		Monthly		Weekly or more			T_1_ (<57·4 mg/d)		T_2_ (57·4–103·4 mg/d		T_3_ (≥103·5 mg/d)		
	Mean	s d	Mean	s d	Mean	s d		Mean	s d	Mean	s d	Mean	s d	
Energy (kJ/d)	2302·9	815	2545·5	771	2852	725	<0·001	2553·8	891	2202·5	687	2380·2	847	<0·001
Protein (g/d)	86·05	33·1	95·03	32·1	96·92	30·6	<0·001	97·7	36·1	81·8	28·0	85·2	33·3	<0·001
Fat (g/d)	94·43	35·5	106·95	35	121·85	34·2	<0·001	105·23	39	91·24	31·2	99·33	38·3	<0·001
Carbohydrate (g/d)	285·3	116	309·9	107	352·1	100	<0·001	312·5	129	271·6	97·8	294·9	116	<0·001
Dietary fibre (g/d)	22·17	9·7	23·90	8·9	24·13	8·35	<0·001	24·67	10·6	21·02	8·24	21·84	9·39	<0·001
Vitamin B_1_ (mg/d)	1·83	0·96	1·91	0·86	1·89	0·74	0·150	2·04	1·07	1·69	0·76	1·77	0·89	<0·001
Vitamin B_2_ (mg/d)	1·84	0·75	1·96	0·68	1·96	0·64	<0·001	2·0	0·82	1·75	0·65	1·83	0·72	<0·001
Vitamin B_3_ (mg/d)	24·07	10·6	26·90	9·8	30·46	9·1	<0·001	27·44	11·7	22·67	8·8	24·8	10·7	<0·001
Vitamin B_5_ (mg/d)	6·1	2·0	6·5	1·9	6·4	1·8	<0·001	6·7	2·2	5·8	1·7	5·9	2·0	<0·001
Vitamin B_6_ (mg/d)	1·94	0·73	2·11	0·71	2·10	0·69	<0·001	2·16	0·79	1·86	0·63	1·98	0·74	<0·001
Vitamin B_12_ (µg/d)	2·87	1·2	3·23	1·2	3·1	1·3	<0·001	3·23	1·4	2·8	1·1	2·8	1·3	<0·001
Folate (µg/d)	569·3	241	599	225	586·7	199	0·028	616·3	270	533·9	194	567·7	233	<0·001
Fe (mg/d)	17·32	7·6	18·55	7·1	18·73	6·4	<0·001	19·36	8·4	16·24	6·1	17·09	7·4	<0·001
Ca (mg/d)	974·86	566	1007·4	494	989·42	427	0·467	1080·9	615	912·2	480	932·2	518	<0·001
Zn (mg/d)	10·84	3·9	11·87	3·8	11·72	3·6	<0·001	12·09	4·2	10·36	3·3	10·71	3·9	<0·001
Tryptophan (µmol/l)	0·68	0·28	0·75	0·27	0·75	0·25	<0·001	0·76	0·31	0·65	0·23	0·67	0·27	<0·001

* Obtained from ANOVA.

Crude and multivariable-adjusted OR and 95 % CI for symptoms of psychological disorders across categories of coffee and caffeine intake are present in Table 3. In the fully adjusted model, individuals who consumed coffee weekly or more had a lower odds of symptoms of depression (OR 0·67; 95 % CI (0·46, 0·96)) and symptoms of anxiety (OR 0·57; 95 % CI (0·34, 0·95)) than those who did not consume coffee. However, we found no significant relationship between coffee intake and odds of symptoms of psychological distress (OR 0·98; 95 % CI (0·68, 1·42)). When possible confounders were considered, we observed no significant association between caffeine intake and odds of symptoms of depression (OR 0·94; 95 % CI (0·75, 1·16)), symptoms of anxiety (OR 0·90; 95 % CI (0·67, 1·20)) and symptoms of psychological distress (OR 1·13; 95 % CI (0·89, 1·42)).

**Table 3:** Crude and multivariable-adjusted OR and 95 % CI for symptoms of psychological disorders across categories of coffee and caffeine intake*

	Coffee intake					*P*_for trend_	Caffeine intake					*P*_for trend_
	None	Monthly		Weekly or more			T_1_ (<57·4 mg/d)	T_2_ (57·4–103·4 mg/d)		T_3_ (≥103·5 mg/d)		
		OR	95 % CI	OR	95 % CI			OR	95 % CI	OR	95 % CI	
Symptoms of depression												
Crude	1·00	0·98	0·79, 1·22	0·82	0·61, 1·09	0·23	1·00	1·06	0·88, 1·28	1·02	0·84, 1·22	0·84
Model I†	1·00	0·94	0·74, 1·19	0·82	0·60, 1·12	0·22	1·00	1·00	0·82, 1·23	1·00	0·82, 1·22	0·97
Model II‡	1·00	1·00	0·78, 1·29	0·87	0·63, 1·20	0·51	1·00	1·04	0·85, 1·29	1·02	0·83, 1·26	0·80
Model III§	1·00	0·97	0·76, 1·25	0·69	0·48, 0·99	0·08	1·00	1·02	0·83, 1·26	0·96	0·77, 1·18	0·71
Model IV‖	1·00	0·97	0·75, 1·26	0·67	0·46, 0·96	0·07	1·00	1·02	0·82, 1·27	0·94	0·75, 1·16	0·57
Symptoms of anxiety												
Crude	1·00	0·96	0·71, 1·29	0·82	0·55, 1·21	0·34	1·00	0·98	0·77, 1·25	0·97	0·76, 1·24	0·82
Model I	1·00	0·93	0·67, 1·28	0·79	0·52, 1·21	0·28	1·00	0·96	.074, 1·26	0·99	0·76, 1·29	0·96
Model II	1·00	1·02	0·73, 1·43	0·81	0·52, 1·28	0·50	1·00	1·01	0·77, 1·33	1·04	0·79, 1·37	0·75
Model III	1·00	1·00	0·71, 1·41	0·61	0·37, 1·01	0·12	1·00	0·98	0·74, 1·29	0·94	0·71, 1·25	0·70
Model IV	1·00	0·98	0·69, 1·39	0·57	0·34, 0·95	0·07	1·00	0·94	0·71, 1·25	0·90	0·67, 1·20	0·48
Symptoms of psychological distress												
Crude	1·00	0·94	0·74, 1·20	1·04	0·78, 1·40	0·94	1·00	1·08	0·88, 1·32	1·12	0·92, 1·37	0·24
Model I	1·00	0·94	0·72, 1·21	1·05	0·77, 1·44	0·93	1·00	1·04	0·83, 1·29	1·17	0·94, 1·45	0·14
Model II	1·00	0·98	0·75, 1·28	1·12	0·81, 1·55	0·57	1·00	1·06	0·84, 1·33	1·26	1·01, 1·57	0·03
Model III	1·00	0·99	0·75, 1·29	1·05	0·73, 1·50	0·84	1·00	1·03	0·82, 1·29	1·18	0·94, 1·47	0·14
Model IV	1·00	0·99	0·75, 1·31	0·98	0·68, 1·42	0·95	1·00	0·99	0·78, 1·25	1·13	0·89, 1·42	0·28

* Data are OR (95 % CI).

† Model I: adjusted for age, sex and energy intake.

‡ Model II: additionally, adjusted for marital status, education, antidepressant use, vitamin supplements use, smoking status, physical activity and existence of a chronic condition.

§ Model III: additionally, adjusted for dietary fibre, *n*-3, vitamin B_1_, vitamin B_2_, vitamin B_3_, vitamin B_5_, vitamin B_6_, vitamin B_12_, folate and tryptophan.

‖ Model IV: additionally, adjusted for BMI.

Gender-stratified crude and multivariable-adjusted OR and 95 % CI for symptoms of psychological disorders across categories of coffee and caffeine intake are indicated in Table 4. We observed no significant relationship between coffee intake and odds of symptoms of depression and anxiety in men. In the crude model, there was a significant positive association between coffee intake and odds of symptoms of psychological distress (OR 1·76; 95 % CI (1·07, 2·92)) in men. This association remained significant after adjustment for energy intake and age (OR 1·91; 95 % CI (1·08, 3·38)). However, this significant relationship disappeared in the fully adjusted model (OR 1·63; 95 % CI (0·82, 3·23)). We observed no significant association between coffee consumption and odds of symptoms of depression (OR 0·70; 95 % CI (0·46, 1·07)), symptoms of anxiety (OR 0·66; 95 % CI (0·37, 1·19)) and symptoms of psychological distress (OR 0·83; 95 % CI (0·54, 1·29)) in women. In addition, we found no significant association between caffeine intake and odds of symptoms of depression, anxiety and psychological distress in either gender.

**Table 4:** Gender-stratified crude and multivariable-adjusted OR and 95 % CI for symptoms of psychological disorders across categories of coffee and caffeine intake

	Coffee intake					*P*_for trend_	Caffeine intake					*P*_for trend_
	None	Monthly		Weekly or more			T_1_ (<57·4 mg/d)	T_2_ (57·4–103·4 mg/d)		T_3_ (≥103·5 mg/d)		
		OR	95 % CI	OR	95 % CI			OR	95 % CI	OR	95 % CI	
Men												
Symptoms of depression												
Crude	1·00	1·33	0·92, 1·91	0·88	0·51, 1·52	0·68	1·00	1·21	0·88, 1·68	1·08	0·78, 1·48	0·63
Model I	1·00	1·32	0·88, 1·99	0·88	0·47, 1·66	0·69	1·00	1·19	0·81, 1·73	1·09	0·76, 1·56	0·64
Model II	1·00	1·36	0·89, 2·08	0·80	0·41, 1·52	0·85	1·00	1·16	0·79, 1·72	1·02	0·70, 1·48	0·93
Model III	1·00	1·26	0·82, 1·95	0·65	0·32, 1·31	0·68	1·00	1·12	0·76, 1·66	0·96	0·66, 1·40	0·81
Model IV	1·00	1·13	0·72, 1·77	0·64	0·31, 1·33	0·50	1·00	1·17	0·78, 1·74	0·89	0·60, 1·33	0·55
Symptoms of anxiety												
Crude	1·00	1·57	0·95, 2·58	0·92	0·41, 2·07	0·45	1·00	0·92	0·57, 1·49	1·08	0·69, 1·69	0·71
Model I	1·00	1·71	0·98, 3·00	0·92	0·35, 2·40	0·40	1·00	1·08	0·60, 1·93	1·45	0·86, 2·46	0·15
Model II	1·00	1·89	1·04, 3·41	0·67	0·24, 1·89	0·67	1·00	1·08	0·59, 1·99	1·28	0·73, 2·24	0·36
Model III	1·00	1·69	0·93, 3·09	0·42	0·13, 1·28	0·67	1·00	1·03	0·56, 1·90	1·15	0·65, 2·03	0·60
Model IV	1·00	1·44	0·76, 2·73	0·33	0·98, 1·17	0·40	1·00	1·01	0·54, 1·89	1·09	0·60, 1·96	0·76
Symptoms of psychological distress												
Crude	1·00	1·46	0·99, 2·15	1·76	1·07, 2·92	0·007	1·00	1·18	0·82, 1·70	1·34	0·95, 1·89	0·08
Model I	1·00	1·47	0·96, 2·52	1·91	1·08, 3·38	0·008	1·00	1·15	0·76, 1·74	1·44	0·99, 2·11	0·05
Model II	1·00	1·52	0·97, 2·39	1·70	0·93, 3·10	0·02	1·00	1·11	0·72, 1·72	1·38	0·92, 2·05	0·10
Model III	1·00	1·49	0·94, 2·36	1·73	0·91, 3·29	0·03	1·00	1·08	0·70, 1·68	1·30	0·87, 1·94	0·19
Model IV	1·00	1·39	0·86, 2·52	1·63	0·82, 3·23	0·07	1·00	1·07	0·68, 1·68	1·15	0·75, 1·75	0·50
Women												
Symptoms of depression												
Crude	1·00	0·84	0·63, 1·11	0·74	0·52, 1·04	0·04	1·00	0·95	0·75, 1·19	0·99	0·78, 1·25	0·97
Model I	1·00	0·79	0·59, 1·07	0·81	0·57, 1·17	0·87	1·00	0·95	0·74, 1·21	0·96	0·75, 1·23	0·79
Model II	1·00	0·87	0·64, 1·18	0·92	0·64, 1·34	0·48	1·00	1·00	0·78, 1·29	1·02	0·79, 1·31	0·86
Model III	1·00	0·85	0·63, 1·16	0·73	0·48, 1·11	0·10	1·00	0·98	0·76, 1·26	0·93	0·72, 1·21	0·63
Model IV	1·00	0·91	0·66, 1·24	0·70	0·46, 1·07	0·11	1·00	0·96	0·74, 1·24	0·94	0·72, 1·22	0·64
Symptoms of anxiety												
Crude	1·00	0·77	0·53, 1·16	0·74	0·47, 1·15	0·08	1·00	0·95	0·71, 1·27	0·93	0·69, 1·25	0·65
Model I	1·00	0·72	0·49, 1·07	0·78	0·48, 1·25	0·11	1·00	0·94	0·70, 1·27	0·87	0·64, 1·19	0·39
Model II	1·00	0·79	0·52, 1·19	0·86	0·52, 1·43	0·34	1·00	0·99	0·73, 1·36	0·95	0·68, 1·31	0·76
Model III	1·00	0·79	0·52, 1·21	0·70	0·40, 1·24	0·14	1·00	0·94	0·69, 1·29	0·83	0·60, 1·16	0·29
Model IV	1·00	0·83	0·55, 1·27	0·66	0·37, 1·19	0·13	1·00	0·90	0·65, 1·23	0·79	0·56, 1·11	0·18
Symptoms of psychological distress												
Crude	1·00	0·75	0·56, 1·02	0·78	0·55, 1·12	0·06	1·00	0·99	0·77, 1·27	1·03	0·81, 1·33	0·76
Model I	1·00	0·75	0·54, 1·03	0·85	0·59, 1·24	0·15	1·00	1·00	0·77, 1·29	1·06	0·82, 1·37	0·63
Model II	1·00	0·78	0·56, 1·09	0·98	0·67, 1·44	0·50	1·00	1·03	0·79, 1·34	1·19	0·91, 1·56	0·19
Model III	1·00	0·78	0·56, 1·10	0·87	0·57, 1·34	0·27	1·00	0·98	0·75, 1·29	1·09	0·83, 1·44	0·51
Model IV	1·00	0·83	0·59, 1·17	0·83	0·54, 1·29	0·26	1·00	0·93	0·71, 1·22	1·08	0·81, 1·43	0·58

Model I: adjusted for age and energy intake.

Model II: additionally, adjusted for marital status, education, antidepressant use, vitamin supplements use, smoking status, physical activity and existence of a chronic condition.

Model III: additionally, adjusted for dietary fibre, *n*-3, vitamin B_1_, vitamin B_2_, vitamin B_3_, vitamin B_5_, vitamin B_6_, vitamin B_12_, folate and tryptophan.

Model IV: additionally, adjusted for BMI.

BMI-stratified crude and multivariable-adjusted OR and 95 % CI for symptoms of psychological across categories of coffee and caffeine consumption are presented in Table 5. In the crud model, we found that individuals with BMI < 25 kg/m^2^ who consumed coffee weekly or more had a lower risk of symptoms of depression (OR 0·67; 95 % CI (0·45, 0·99)). This significant association remained significant after adjustment for age, sex and energy intake. However, after further adjustment for possible confounders, this relationship became non-significant (OR 0·66 95 % CI (0·41, 1·06)). Taking potential confounders into account, we observed a significant inverse association between coffee consumption and symptoms of anxiety among individuals with BMI < 25 kg/m^2^ (OR 0·39; 95 % CI (0·18, 0·82)). There was no significant relationship between coffee consumption and symptoms of psychological distress in subjects with BMI < 25 kg/m^2^. We found no significant association between coffee intake and symptoms of depression, anxiety and psychological distress among individuals with BM ≥ 25 kg/m^2^. Neither in overweight (BMI ≥ 25 kg/m^2^) nor in normal weight (BMI < 25 kg/m^2^) participants, we failed to find a significant association between caffeine intake and odds of symptoms of depression, anxiety and psychological distress.

**Table 5:** BMI-stratified crude and multivariable-adjusted OR and 95 % CI for symptoms of psychological disorders across categories of coffee and caffeine intake

	Coffee intake					*P*_for trend_	Caffeine intake					*P*_for trend_
	None	Monthly		Weekly or more			T_1_ (<57·4 mg/d)	T_2_ (57·4–103·4 mg/d)		T_3_ (≥103·5 mg/d)		
		OR	95 % CI	OR	95 % CI			OR	95 % CI	OR	95 % CI	
BMI < 25 kg/m^2^												
Symptoms of depression												
Crude	1·00	0·87	0·65, 1·18	0·67	0·45, 0·99	0·04	1·00	0·87	0·67, 1·12	0·91	0·70, 1·17	0·45
Model I	1·00	0·90	0·64, 1·25	0·65	0·42, 0·99	0·05	1·00	0·84	0·63, 1·10	0·91	0·69, 1·20	0·51
Model II	1·00	0·95	0·67, 1·33	0·71	0·46, 1·10	0·16	1·00	0·88	0·66, 1·17	0·92	0·68, 1·22	0·56
Model III	1·00	0·95	0·67, 1·35	0·66	0·41, 1·06	0·13	1·00	0·85	0·64, 1·14	0·86	0·64, 1·16	0·33
Symptoms of anxiety												
Crude	1·00	0·86	0·57, 1·30	0·52	0·28, 0·95	0·03	1·00	0·85	0·60, 1·20	0·92	0·65, 1·30	0·62
Model I	1·00	0·85	0·54, 1·32	0·47	0·25, 0·92	0·02	1·00	0·91	0·63, 1·31	0·98	0·67, 1·42	0·91
Model II	1·00	0·88	0·55, 1·41	0·48	0·24, 0·96	0·04	1·00	0·97	0·66, 1·43	1·04	0·70, 1·54	0·82
Model III	1·00	0·89	0·55, 1·43	0·39	0·18, 0·82	0·02	1·00	0·92	0·62, 1·35	0·93	0·62, 1·39	0·73
Symptoms of psychological distress												
Crude	1·00	0·88	0·63, 1·21	0·83	0·56, 1·25	0·28	1·00	1·00	0·76, 1·32	1·04	0·79, 1·38	0·73
Model I	1·00	0·93	0·65, 1·32	0·85	0·55, 1·31	0·43	1·00	0·94	0·70, 1·26	1·04	0·77, 1·40	0·77
Model II	1·00	0·92	0·64, 1·34	0·90	0·58, 1·40	0·58	1·00	0·96	0·71, 1·31	1·10	0·81, 1·49	0·54
Model III	1·00	0·96	0·66, 1·39	0·95	0·59, 1·54	0·80	1·00	0·94	0·69, 1·28	1·07	0·78, 1·46	0·67
BMI ≥ 25 kg/m^2^												
Symptoms of depression												
Crude	1·00	1·14	0·82, 1·59	1·01	0·66, 1·56	0·67	1·00	1·39	1·05, 1·85	1·20	0·90, 1·58	0·23
Model I	1·00	1·03	0·71, 1·48	1·14	0·71, 1·83	0·59	1·00	1·31	0·96, 1·78	1·19	0·88, 1·61	0·28
Model II	1·00	1·14	0·78, 1·67	1·18	0·72, 1·96	0·37	1·00	1·32	0·96, 1·82	1·25	0·91, 1·71	0·18
Model III	1·00	1·08	0·73, 1·58	0·83	0·47, 1·46	0·76	1·00	1·30	0·94, 1·80	1·16	0·84, 1·60	0·37
Symptoms of anxiet												
Crude	1·00	1·05	0·68, 1·63	1·13	0·65, 1·95	0·63	1·00	1·11	0·77, 1·59	0·94	0·66, 1·35	0·75
Model I	1·00	0·98	0·60, 1·59	1·29	0·70, 2·38	0·51	1·00	1·01	0·68, 1·50	0·96	0·64, 1·42	0·83
Model II	1·00	1·16	0·69, 1·95	1·33	0·69, 2·58	0·32	1·00	1·02	0·68, 1·55	1·01	0·67, 1·53	0·94
Model III	1·00	1·13	0·67, 1·91	0·92	0·43, 1·96	0·93	1·00	1·02	0·67, 1·54	0·92	0·60, 1·40	0·69
Symptoms of psychological distress												
Crude	1·00	1·05	0·73, 1·52	1·30	0·83, 2·03	0·26	1·00	1·16	0·85, 1·58	1·22	0·90, 1·65	0·19
Model I	1·00	0·97	0·66, 1·44	1·40	0·86, 2·29	0·28	1·00	1·15	0·82, 1·62	1·35	0·98, 1·88	0·06
Model II	1·00	1·08	0·72, 1·62	1·50	0·90, 2·49	0·13	1·00	1·15	0·81, 1·64	1·50	1·07, 2·11	0·01
Model III	1·00	1·05	0·69, 1·59	1·20	0·68, 2·13	0·52	1·00	1·13	0·79, 1·60	1·35	0·96, 1·91	0·07

Model I: adjusted for age, sex and energy intake.

Model II: additionally, adjusted for marital status, education, antidepressant use, vitamin supplements use, smoking status, physical activity and existence of a chronic condition.

Model III: additionally, adjusted for dietary fibre, *n*-3, vitamin B_1_, vitamin B_2_, vitamin B_3_, vitamin B_5_, vitamin B_6_, vitamin B_12_, folate and tryptophan.

## Discussion

In this cross-sectional study, we evaluated the association between coffee and caffeine intake and odds of symptoms of psychological disorders. We found that those who consumed coffee weekly or more had a signiﬁcant lower chance of symptoms of anxiety and depression. However, we observed no significant relationship between coffee consumption and odds of symptoms of psychological distress. In addition, we observed no signiﬁcant association between caffeine intake and symptoms of psychological disorders, neither in the whole population nor in the stratified analyses.

Our findings on the link between coffee intake and symptoms of depression were in agreement with previous studies. A prospective cohort study found that coffee, but not caffeine, intake may reduce the risk of depression^(30)^. In addition, findings from a cross-sectional study revealed that coffee consumption was associated with a low prevalence of depressive symptoms; however, there was no significant association between caffeine consumption and prevalence of depressive symptoms^(31)^. Similar findings were also reported from Japan, where individuals who consumed ≥2 cups/d coffee had a lower prevalence of depressive symptoms than those who consumed <1 cup/d; however, this association was not seen for caffeine intake^(32)^. The protective association of coffee consumption against risk of depression was also seen in a cross-sectional study in Korea^(33)^. In contrast to ours, some studies reached a positive association between coffee consumption and psychological disorders. For instance, in a cross-sectional study, female participants who drank ≥4 cups/d of any form of coffee had an increased likelihood of having major depression compared with non-coffee drinkers; however, there was no relationship between <4 cups/d coffee consumption and odds of depression^(15)^. Another study in Korea revealed that a greater intake of caffeine was positively associated with depression^(16)^. Different findings in different studies might be explained by the discrepancy in subject characteristics and different sample sizes as well as different definitions of depression used. In addition, most studies did not adjust for many potential confounders, especially for dietary intakes that can confound the association between coffee and caffeine intake and symptoms of psychological disorders. Because there was so little information about anxiety, as in a cross-sectional study in the USA found no significant association between coffee consumption and anxiety symptoms^(14)^, we focused on symptoms of depression to compare findings.

We found no significant association between coffee or caffeine consumption and symptoms of psychological disorders in either gender. Contrary to our findings, previous studies showed a significant inverse relationship between coffee and caffeine consumption and risk of depression in women^(8)^. In addition, some other studies showed that coffee and caffeine intake increased the risk of anxiety in males, but not in females^(34,35)^. The results of the relationship between coffee and caffeine consumption and psychological disorders in men and women are controversial. Further studies are needed to examine the gender discrepancy in this association.

The reason why we did not observe any significant association between caffeine intake and symptoms of psychological disorders might be explained by the low caffeine intake in the study population. In our study, the average caffeine intake was 98·1 mg/d. However, in previous studies that observed a significant relationship, the average consumption was higher than our study. In a study conducted among university students, the average intake of caffeine was 268 mg/d. Individuals who consumed high caffeine (more than 400 mg/d) in that study had an elevated chance for anxiety and psychological distress^(36)^. In the study of Lucas 2011, which reported an inverse relationship between caffeine consumption and risk of depression, people in the highest category consumed ≥550 mg/d^(8)^. However, in our study, those in the highest category of caffeine consumed an average of 185·3 mg/d, which was far lower than the previous studies. Therefore, we assumed that the low amount of caffeine intake, which is due to the food culture of Iranians, might justify the lack of finding a significant relationship.

In our study, when we performed gender-stratified analysis, the associations disappeared. This might be explained by the presence of some gender-dependent confounders that we failed to control for them in our analysis. Another point that might provide some reasons for this finding is the number of people with symptoms of psychological disorders that would be low in the gender-stratified analysis, compared to the combined analysis on men and women together. Such a low number of people with the outcome would result in wide CI of OR that would in turn result in a non-significant association.

In BMI-stratified analyses, we found no significant association between caffeine intake and symptoms of psychological disorders. In line with our study, a cross-sectional study reported no significant association between caffeine intake and odds of depression in BMI-stratified analyses^(11)^. In this study, a significant inverse association was observed between coffee consumption and odds of anxiety in subjects with BMI < 25 kg/m^2^. This finding might be explained by the fact that being overweight might mask the favourable association of coffee intake and symptoms of depression and anxiety in obese people due to the elevated inflammation that might be aroused from accumulated body fat.

The mechanisms through which coffee intake might influence psychological health are largely unknown; however, these effects can be attributed to the presence of antioxidant substances with potentially beneficial properties, for example, chlorogenic acid, flavonoids, melanoidins and trigonelline in coffee^(37,38)^. In a recent study, chlorogenic acid protects pheochromocytoma (PC12) cells against corticosterone-induced neurotoxicity related to inhibition of autophagy and apoptosis and, in turn, may alleviate depression^(39)^. Neuroprotective, antioxidant, anti-inflammatory and anti-apoptotic properties have also been found in some coffee phenolic acid compounds such as caffeic acid, chlorogenic acid and ferulic acid^(40)^. An animal study found that caffeic acid and caffeine in coffee exhibit anxiolytic and antidepressant effects by protecting inflammatory markers^(41)^. Also, the phenolic acids in coffee cross the blood–brain barrier and are present in the cerebrospinal fluid and could possibly affect brain health^(42)^. In this regard, a recent study showed that a higher intake of dietary phenolic acid was associated with a significant reduction in cognitive status^(43)^.

Some studies have attributed the effects of coffee consumption to its caffeine content^(8)^; however, we failed to find any significant association between caffeine intake and symptoms of depression. This shows that factors other than caffeine might be involved in the protective association of coffee against symptoms of psychological disorders.

Several strengths in this study make the findings interesting. The large sample size of the study and considering a wide range of potential confounders in the statistical analysis are among the strengths of this study. Although, our study has some limitations which must be noted. The cross-sectional design of this study would not allow us to confer a causal relationship between coffee and caffeine intake and symptoms of psychological disorders; therefore, more prospective studies are required to support our results. Furthermore, causality in cross-sectional studies cannot be inferred. Individuals who are anxious or depressed consume more coffee, which is a comfort drink. Such mental disorders might lead to higher consumption of coffee, chocolate, sweets, etc. Although we controlled the analyses for many potential confounders, residual confounding cannot be excluded. Coffee consumption might be affected by the health condition of some participants. In addition, the pattern of consumption of these kinds of comfort foods or drinks is strongly associated with other lifestyle characteristics that might not be captured in our analyses. Moreover, using self-reported questionnaires could be considered as another limitation of this study which might lead to misclassifications of participants in terms of exposure and outcome. Although we used a validated FFQ for the assessment of dietary intakes, the questionnaire was not validated for coffee and caffeine intake. In addition, participants of our study were healthcare workers, which might be health-conscious and have different characteristics than the general population. Moreover, in this study, detailed information about the type of coffee and its concentration was not collected and we did not investigate the association of different types of coffee with symptoms of psychological disorders due to a lack of data. Finally, we considered energy intakes outside the range of 800–4200 kJ/d as an exclusion criterion in the whole study population; however, some investigators have used different cut-off points to define energy under- and over-reported among men and women^(12)^. When we used the suggested cut-off point of 500–3500 kJ/d for women, we had to further exclude 187 women. However, our main findings did not differ even with excluding these women from the study.

In conclusion, compared with lack of coffee intake, weekly or more coffee consumption might be correlated with symptoms of depression and anxiety. No significant correlation was observed between coffee intake and symptoms of psychological distress. Caffeine intake was not correlated with symptoms of psychological disorders.
